# Dissociation in performance of children with ADHD and high-functioning autism on a task of sustained attention

**DOI:** 10.1016/j.neuropsychologia.2007.02.019

**Published:** 2007

**Authors:** Katherine A. Johnson, Ian H. Robertson, Simon P. Kelly, Timothy J. Silk, Edwina Barry, Aoife Dáibhis, Amy Watchorn, Michelle Keavey, Michael Fitzgerald, Louise Gallagher, Michael Gill, Mark A. Bellgrove

**Affiliations:** a School of Psychology and Trinity College Institute of Neuroscience, Trinity College Dublin, Dublin 2, Ireland; b School of Medicine and Health Sciences and Trinity College Institute of Neuroscience, Trinity College Dublin, Dublin 2, Ireland; c Cognitive Neurophysiology Laboratory, Nathan S. Kline Institute, Orangeburg, NY 10962, United States; d Howard Florey Institute and Centre for Neuroscience, University of Melbourne, Australia; e Academic Child Psychiatry Unit, Department of Pediatrics, University of Melbourne, Australia; f Department of Psychology, Monash University, Australia; g Cognitive Neuroscience Laboratory, School of Psychology and Queensland Brain Institute, University of Queensland, Brisbane, Australia

**Keywords:** Response time, Fast Fourier transform, Variability, Arousal, Response inhibition, Executive function

## Abstract

Attention deficit hyperactivity disorder (ADHD) and autism are two neurodevelopmental disorders associated with prominent executive dysfunction, which may be underpinned by disruption within fronto-striatal and fronto-parietal circuits. We probed executive function in these disorders using a sustained attention task with a validated brain-behaviour basis. Twenty-three children with ADHD, 21 children with high-functioning autism (HFA) and 18 control children were tested on the Sustained Attention to Response Task (SART). In a fixed sequence version of the task, children were required to withhold their response to a predictably occurring no-go target (3) in a 1–9 digit sequence; in the random version the sequence was unpredictable. The ADHD group showed clear deficits in response inhibition and sustained attention, through higher errors of commission and omission on both SART versions. The HFA group showed no sustained attention deficits, through a normal number of omission errors on both SART versions. The HFA group showed dissociation in response inhibition performance, as indexed by commission errors. On the Fixed SART, a normal number of errors was made, however when the stimuli were randomised, the HFA group made as many commission errors as the ADHD group. Greater slow-frequency variability in response time and a slowing in mean response time by the ADHD group suggested impaired arousal processes. The ADHD group showed greater fast-frequency variability in response time, indicative of impaired top-down control, relative to the HFA and control groups. These data imply involvement of fronto-parietal attentional networks and sub-cortical arousal systems in the pathology of ADHD and prefrontal cortex dysfunction in children with HFA.

## 1 Introduction

Autism and attention deficit hyperactivity disorder (ADHD) are two common, largely genetic, childhood-onset psychiatric disorders affecting key fronto-striatal and fronto-parietal circuits that are important for executive function (Courchesne & Pierce, 2005; Filipek et al., 1997; Mostofsky, Cooper, Kates, Denckla, & Kaufmann, 2002; Schmitz et al., 2006). These two disorders differ substantially in symptom presentation, but they also share a number of important features (Sturm, Fernell, & Gillberg, 2004). Despite the exclusion of one disorder in the formal diagnosis of the other, there appears to be a degree of comorbidity (or a sharing of symptoms) between the two disorders (Goldstein & Schwebach, 2004; Holtmann, Bölte, & Poustka, 2005; Stahlberg, Soderstrom, Rastam, & Gillberg, 2004). Both disorders have a strong genetic component to their aetiology, with heritability estimates of 0.9 for autism and 0.7 for ADHD (Baron-Cohen & Belmonte, 2005; Faraone et al., 2005); indeed there is preliminary evidence of genetic linkage in autism and ADHD at chromosomal locations 2q24 and 16p13 (Fisher et al., 2002; International Molecular Genetic Study of Autism Consortium, 2001). Executive dysfunction is associated with both autism (Geurts, Verte, Oosterlaan, Roeyers, & Sergeant, 2004) and ADHD (Willcutt, Doyle, Nigg, Faraone, & Pennington, 2005). Some very specific aspects of executive dysfunction have recently been proposed as endophenotypes in genetic association studies in ADHD: one in particular is variability in response time (RT) on tasks that measure sustained attention capabilities (Castellanos & Tannock, 2002; Kuntsi & Stevenson, 2001). Increasingly, response time variability is being seen as a legitimate marker of brain pathology, particularly in the frontal areas (Bellgrove, Hester, & Garavan, 2004; MacDonald, Nyberg, & Bäckman, 2006; Stuss, Murphy, Binns, & Alexander, 2003). It would be extremely useful to know if a candidate endophenotype is able to distinguish between two neurodevelopmental disorders. Dissociating autism and ADHD on a task of executive function may help to define disorder-specific markers for use in genetic association studies. In this context we sought to determine whether children with ADHD and autism differed on archetypal executive functions, that of sustained attention and response inhibition, and particularly if variability in RT specifically differentiated these two groups.

Sustained attention is the endogenous ability to mindfully and consciously process stimuli, whose non-arousing qualities would otherwise lead to habituation and distraction (Robertson, Manly, Andrade, Baddeley, & Yiend, 1997). The ability to sustain attention to a task and produce an appropriate response entails the functioning of the fronto-parietal circuit. The right dorsolateral prefrontal cortex and the right inferior parietal cortex are activated during sustained attention tasks (Coull, Frackowiak, & Frith, 1998; Coull, Frith, Frackowiak, & Grasby, 1996; Fassbender et al., 2004; O’Connor, Manly, Robertson, Hevenor, & Levine, 2004; Pardo, Fox, & Raichle, 1991). In addition, the anterior cingulate, basal ganglia and thalamus are likely to be involved in regulating and co-ordinating appropriate responses during attentional tasks (Bradshaw, 2001; Bush et al., 1999; Coull et al., 1998; Konrad, Neufang, Hanisch, Fink, & Herpertz-Dahlmann, 2006). Finally, areas of the midbrain involved in arousal, including the reticular formation and the locus coeruleus, may sub-serve the ability to maintain attention to a task over time (Coull, 1998; Sturm et al., 1999).

There is evidence of anatomical and physiological dysfunction in the fronto-parietal and fronto-striatal networks in ADHD. Researchers have found bilateral reductions in prefrontal volume (Castellanos et al., 2002; Filipek et al., 1997; Mostofsky et al., 2002; Sowell et al., 2003), reduced white matter in the parietal–occipital regions (Filipek et al., 1997) and increased grey matter in the inferior parietal cortices (Sowell et al., 2003). Subcortically, there is reduced anatomical volume of the caudate nucleus, putamen and cerebellum (Castellanos et al., 1994, 1996, 2002). Functionally, the dorsal anterior cingulate has been found to be hypoactive (Bush et al., 1999; Durston et al., 2003; Rubia et al., 1999) and functioning of the fronto-striatal circuit (Schweitzer et al., 2003) and prefrontal cortices are abnormal (Bush et al., 1999; Durston et al., 2003; Rubia et al., 1999). Dysfunction within the parietal lobe, particularly of the right-hemisphere, has been noted in a number of recent reports (Booth et al., 2005; Silk et al., 2005). Please refer to (Bush, Valera, & Seidman, 2005) for a recent review.

There is also anatomical and physiological evidence of fronto-striatal (Abell et al., 1999; Carper & Courchesne, 2005; Courchesne et al., 2001; McAlonan et al., 2005; Silk et al., 2006; Voelbel, Bates, Buckman, Pandina, & Hendren, 2006) and fronto-parietal dysfunction in autism (Just, Cherkassky, Keller, Kana, & Minshew, in press; Schmitz et al., 2006). Anatomical research suggests that children with autism have abnormally large frontal lobe volumes (Carper & Courchesne, 2000, 2005; Courchesne et al., 2001; Schmitz et al., 2006) that might reflect a lack of synaptogenesis early in life (Belmonte et al., 2004). There appears to be diminished grey matter in fronto-striatal and parietal networks (Courchesne et al., 2001) although there is some debate as to whether anatomical abnormalities exist in the parietal lobes (Abell et al., 1999; Courchesne, Press, & Yeung-Courchesne, 1993; Koshino et al., 2005; McAlonan et al., 2002; Schmitz et al., 2006). Functionally, people with autism show a decrease in regional cerebral blood flow (rCBF) in left prefrontal cortices (Ohnishi et al., 2000). During response inhibition tasks, adults with autism show increased activation of the frontal and parietal cortices, compared with controls, despite showing normal behavioural performance on these tasks (Schmitz et al., 2006). Greater left hemisphere activity in the inferior and orbitofrontal cortices may indicate an alternative, compensatory mechanism in adult autism (Schmitz et al., 2006). The recruitment of additional areas of the brain to aid in task performance and to off-set the negative effects of a deficient network has been illustrated in recent studies, including those investigating aged individuals (Nielson, Langenecker, & Garavan, 2002) and first-degree relatives of patients with schizophrenia (Yeap et al., 2006). Executive dysfunction in ADHD and autism may be related to the compromised workings of the fronto-striatal and fronto-parietal circuits.

Equivocal evidence exists for sustained attention deficits in both ADHD and autism, although a greater amount of research has been directed at elucidating sustained attention deficits in ADHD, when compared with autism (e.g. Bellgrove, Hawi, Gill, & Robertson, 2006; Manly et al., 2001). Some researchers have argued that in order to show a deficit in sustained attention, a time-on-task effect for the number of errors must be shown (van den Bergh et al., 2006). In ADHD research, a number of studies have failed to show time-on-task effects with children with ADHD (Stins et al., 2005; van der Meere, Wekking, & Sergeant, 1991), although other studies have demonstrated significant deficits in performance over the duration of the task (Epstein et al., 2003; Heinrich et al., 2001; Johnson et al., 2007). Only a few studies have investigated sustained attention in children with autism. Most studies have used the continuous performance task (CPT) and reported intact sustained attention in autism. Unfortunately, RT was not recorded in three studies (Buchsbaum et al., 1992; Garretson, Fein, & Waterhouse, 1990; Siegel, Nuechterlein, Abel, Wu, & Buchsbaum, 1995), only median RT was analysed in one study (Noterdaeme, Amorosa, Mildenberger, Sitter, & Minow, 2001) and two studies used food or money as obvious (and thus attention-attracting) rewards every time a correct hit was made by the child (Garretson et al., 1990; Pascualvaca, Fantie, Papageorgiou, & Mirsky, 1998). One recent study has directly compared the performance of children with ADHD and HFA on the Integrated Visual and Auditory (IVA) Continuous Performance Test (IVA), a test that combines inattention and impulsivity in the visual and auditory domains (Corbett & Constantine, 2006). Unfortunately, the dependent variables for this task are a combination of re-weighted dependent variables (Corbett & Constantine, 2006). Nevertheless, this study found little difference in performance of the ADHD and HFA groups except on a measure containing elements of impulsivity, consistency of RT and sustained attention (VRCQ) (Corbett & Constantine, 2006). It is difficult to determine which element of the VRCQ was driving this difference in performance between the two groups. Thus, the nature of sustained attention deficits in autism remains to be fully determined. Error rates and RT performance both have the capability of furthering our understanding of sustained attention deficits in children with ADHD, with autism and in control children.

Recently we described a new procedure to analyse RT data to dissociate variability in RT into temporal components of fast (moment-to-moment) and slow variability using a Fast Fourier Transform (FFT) (Johnson et al., 2007), based on the work of Castellanos et al. (2005). The task employed was the Sustained Attention to Response Task (SART), which requires participants to withhold a response to an infrequent target and respond to all other stimuli (Robertson et al., 1997). This task differs substantially from the traditional CPTs, in which participants monitor a stream of stimuli for the occurrence of an infrequent target, which by its very nature has an attention-arousing quality. Instead, the SART tests the ability of the participant to inhibit the automatised act of button pressing when the target appears. *Withholding* to a rare target, as opposed to *responding* to a rare target in CPT tasks, shifts the automatic response set to the non-targets. Successful withholding of the primed response places greater demand on the sustained attention system in order to interrupt the ongoing action (Bellgrove, Hawi, Kirley, Gill, & Robertson, 2005; Robertson et al., 1997). In addition, the SART provides an ample amount of time-series RT data for analysis using the FFT. Different forms of variance can be measured from the FFT spectrum, distinguishing distinct components of RT variability: (1) gradual variability, which has a slow temporal characteristic and (2) trial-to-trial variability, which has a fast temporal characteristic. In contrast, variability measured simply as standard deviation over a task run represents the combined influence of these components but provides no indication of relative contributions. Slow variability is thought to reflect declining arousal over the course of the task, whereas fast variability may reflect fluctuations in top-down attentional control (Johnson et al., 2007).

The SART activates the same right fronto-parietal attentional network (Fassbender et al., 2004; Manly et al., 2003) that appears dysfunctional in ADHD and autism (Giedd, Blumenthal, Molloy, & Castellanos, 2001; McAlonan et al., 2005; Silk et al., 2005). In this study we employed fixed- and random-sequence versions of the SART. The Random SART has a greater response inhibition loading than the Fixed SART, due to the random stimuli presentation. The Fixed SART places a larger endogenous demand upon the sustained attention system, due to the predictability of the stimuli presentation. Errors of commission (responding to the no-go stimuli) on the Fixed SART primarily reflect lapses of sustained attention and to a lesser degree deficits in response inhibition. Commission errors on the Random SART reflect to a greater degree response inhibition deficits, in addition to lapses of sustained attention. Errors of omission (failure to respond to the go-stimuli) in either SART reflect a break from task engagement and thus are reflective of lapsing attention. The current study therefore employed both the fixed and random version of the SART to examine the dissociation between sustained attention and response inhibition in children with ADHD, autism and controls.

The central aim of this study was to assess the ability of children with ADHD, with autism and normal healthy children on these tasks, in order to examine if these groups differed on aspects of sustained attention, response inhibition and response time variability (fast and slow). Based on the previous experimental literature, we hypothesised that the ADHD group would make more errors of commission and omission and show greater fast and slow variability in RT than the control group, on both versions of the SART. Based on anatomical and physiological evidence, we hypothesised that the HFA group, on both versions of the SART, would make more errors of commission and omission and show greater fast and slow variability in RT than the control group and in a similar manner as the ADHD group, but that there may be some evidence of compensatory mechanisms in the measures.

## 2 Method

### 2.1 Participants

Twenty three children with ADHD (3 females), 21 children with HFA (1 female) and 18 control children (3 females) participated in the study (see Table 1). There was no significant difference between the mean ages or in IQ, as measured using four subtests (picture completion, vocabulary, information, block design) of the Weschler Intelligence Scale for Children (Weschler, 1992), between the three groups. The data of 15 of the children with ADHD and 5 controls had previously been published and these children were randomly chosen to match the children with HFA according to age and IQ (Johnson et al., 2007).

Exclusion criteria for participation in the study included known neurological conditions or pervasive developmental disorders (apart from the presently studied disorders for each group), serious head injuries and below average intelligence (below 70 on the WISC-III) (Weschler, 1992). Control children were also excluded if they had first degree relatives with ADHD or HFA. Handedness was measured using the Edinburgh Handedness Inventory (Oldfield, 1971).

The participants with ADHD and HFA were recruited as part of ongoing genetic studies (Gallagher, Hawi, Kearney, Fitzgerald, & Gill, 2004; Kirley et al., 2002). These participants were either referred by consultant psychiatrists or recruited through support groups.

Diagnosis for the participants with ADHD was confirmed by psychiatrists using the parent form of the Child and Adolescent Psychiatric Assessment (CAPA) (Angold et al., 1995). Twenty-two children with ADHD met DSM-IV diagnosis for Combined-type ADHD and one for the Inattentive subtype (American Psychiatric Association, 1995). At the time of testing, all parents of the children with ADHD completed the Conners’ Parent Rating Scale—Revised: Short Version (CPRS-R:S) (Conners, 1997) and all had ADHD Index T-scores greater than 65 (mean 79, S.D. 6). In addition, parents completed the Asperger Syndrome (and high-functioning autism) Diagnostic Interview (ASDI) (Gillberg, Gillberg, Råstram, & Wentz, 2001), either at the time of testing (for 16 children) or retrospectively (for 7 children) approximately 24 months post-testing. The mean of the whole ADHD group on the ASDI was 0.9 (S.D. 1.0). Fifty-six percent of the children with ADHD met diagnostic criteria for oppositional defiant disorder and 13% met diagnostic criteria for conduct disorder. Any stimulant medication was withdrawn for at least 24 h prior to testing. Seventy-four percent of the children with ADHD were stimulant naïve.

Diagnosis for the participants with HFA was confirmed by psychiatrists using the Autism Diagnostic Interview–Revised (ADI-R) (Lord, Rutter, & Le Couteur, 1994) and Autism Diagnostic Observation Schedule–Generic (ADOS-G) (Lord et al., 2000) criteria for autism/autism spectrum disorder. Exclusion criteria included known medical causes of autism, chromosomal abnormalities, or fragile X syndrome. Two participants were on psychotropic medications at the time of the study.^1^ One participant refused to complete the WISC. All parents of the children with HFA completed the ASDI and the CPRS-R:S. For eleven children this was done in a retrospective fashion, approximately 24 months post-testing and for 10 children this was completed at the time of testing. The mean score on the ASDI of the children with HFA was 5.0 (S.D. 0.9). Twelve children (57%) with HFA scored greater than 65 on the ADHD Index (group mean 66, S.D. 12).

The control children were recruited from Dublin schools. Parents of control children completed the CPRS-R:S (Conners, 1997) at the time of testing and all had ADHD Index T-scores less than 60 (mean 45, S.D. 5). Seventeen parents also completed the ASDI, retrospectively, approximately 6 months post-testing (mean 0.1, S.D. 0.2). Consent was obtained from parents of all children and the experimental work was conducted under the approval of local ethical committees in accordance with the Declaration of Helsinki.

### 2.2 Apparatus and procedure

**Fig. 1:**
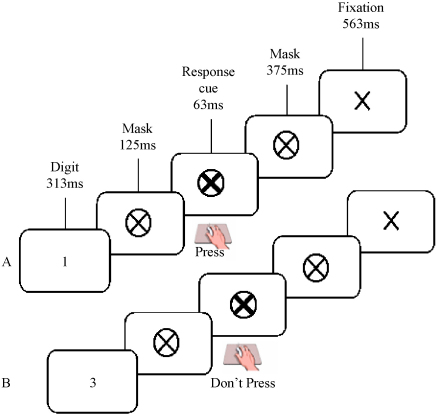
A pictorial representation of the Sustained Attention to Response Task (SART), demonstrating the sequence of events and timings for the SART. Figure depicts (A) a go trial (requiring a response to the presentation of the go-digit 1), and (B) a no-go trial (requiring the withholding of a response to the no-go digit 3). In the Fixed version of the SART, the digits 1–9 are presented within a fixed sequence that is repeated 25 times. In the Random version of the SART, the digits are presented in a pseudo-random order. All participants responded on the response cue.

All participants performed the Fixed and Random versions of the Sustained Attention to Response Task, presented on a laptop computer (Robertson et al., 1997) (please see Fig. 1). In the Fixed version, a repeating fixed sequence of digits (1–9) was presented. In the Random version, the digits appeared in a pseudorandom order. For both versions, a single digit appeared on the screen for 313 ms; a mask was then presented for 125 ms, after which a response cue (a bold cross) appeared for 63 ms, followed by a second mask for 375 ms and a fixation cross for 563 ms. The total inter-stimulus interval was 1439 ms (digit onset to digit onset). Participants were instructed to respond, using a button press, to every digit (go-trial) except ‘3’ (no-go trial). They were asked to respond when the response cue appeared on screen 125 ms after the digit was extinguished, or 438 ms from the start of the trial. The response cue was used to limit the impulsive response style of the ADHD children and to reduce any speed/accuracy trade-offs (Bellgrove et al., 2005). For each of the Fixed and Random SARTs, participants performed 225 trials, representing 25 runs of the 1–9 sequence, lasting approximately 5.5 min. The presentation of the Fixed and Random SARTs was counterbalanced across participants.

### 2.3 Data analysis

For both the Fixed and Random SARTs, errors of commission (responses made on the no-go digit 3) and omission (non-responses on the go-trials) were calculated both for the entire trial (“full-run”) and for the first and second halves of the trial (“half-by-half”). The Mean and standard deviation (S.D.) of the RTs on the go-trials were calculated for the full-run and half-by-half analyses. The sequence of 225 RTs was also analysed using a fast Fourier transform (FFT), following the methodology of Johnson et al. (2007). Grand average FFT spectra were also calculated per group for descriptive purposes.

Data preparation for FFTs: To calculate the FFTs, the RTs for the digit 3 and RTs of less than 100 ms were linearly interpolated from the immediately preceding and following RTs. For the fast-frequency area under the spectra (FFAUS), individual RT data were detrended, subtracting out any linear components, which were analysed separately.

Derivation of FFT spectra: The RT data were analysed according to Welch's averaged, modified periodogram method. The RT data were analysed both over the full-run (225 data points per individual) and in the half-by-half analysis. The full time-series was first divided into 7 segments of 75 data points, with an overlap of 50. Each segment was Hamming-windowed and zero-padded to length 450.^2^ The FFT was then calculated for each segment. For the full-run analyses, the FFT for each segment was averaged across the 7 segments to provide a spectrum per individual. For the half-by-half analysis, the first three FFT segments were averaged in the first half and the last three segments were averaged in the second half. All RT data points were represented in this analysis, due to the 50 data point overlap. Any segments of 75 data points where there were over 10 errors of omission (not necessarily occurring together) were excluded in the FFT. Subsequently, for both the full-run and half-by-half analyses, a small number of participants were excluded (see Table 1).

**Fig. 2:**
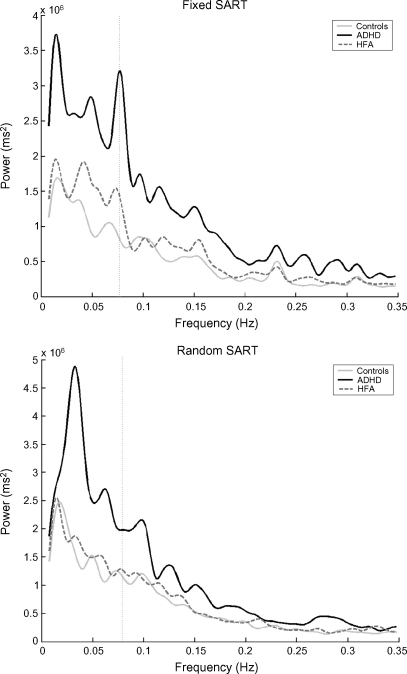
Grand average of the fast Fourier transform (FFT) of the mean response time (RT) data on the Fixed and Random versions of the Sustained Attention to Response Task (SART) for the attention deficit hyperactivity disorder (ADHD), high-functioning autism (HFA) and control groups. The *Y*-axis represents the power of periodic changes in RT data. The *X*-axis represents the different temporal frequencies, in Hertz (Hz). The peak at 0.0772 Hz (reciprocal of 9 digits × 1.439 second inter-stimulus interval and marked by the dotted line) in the Fixed version of the SART is the Principle SART peak, and represents a consistent and distinct pattern of RT performance, such as a slowing in RT in response to digit 1, relative to digit 9 and 2, in preparation for the no-go response on digit 3 (Johnson et al., 2007). This peak is not present in the Random version, due to the random presentation of stimuli. Grand average spectra were calculated per group using the FFT function in MatLab 7.0 (The MathWorks, Natick, Massachusetts).

RT variance may be measured by calculating the area under the spectrum (AUS) over a broad band of interest. The AUS represents a measure of the ‘power’ or overall variance in the signal. The peak power at a particular point in the spectra measures consistency and distinctness of a particular RT pattern. Healthy adult control participants often show a slowing in RT on digit 1 relative to digits 9 and 2 in preparation for the upcoming no-go response on the Fixed SART (Dockree et al., 2004). If this average pattern is consistently reproduced on every 1–9 sequence, a peak in the spectra at 0.0772 Hz is found (reciprocal of 9 digits × 1.439 second inter-stimulus interval) (see dotted line in Fig. 2). This peak was used as a marker to divide the variability into two components. The fast-frequency area under the spectra encompassed all sources of variability faster than once per SART cycle (0.0772 Hz) (area under curve to the right of dotted line in Fig. 2). Any trial-to-trial variability was captured in this calculation. The slow-frequency AUS (SFAUS) encompassed all sources of variability slower than once per SART cycle (area under curve to the left of dotted line in Fig. 2). Any variability that occurred over any time period greater than one SART cycle was captured in this calculation. To ensure that all low frequencies were encompassed in the SFAUS, the time series was not divided into segments. Any RT time series where there were greater than 5 errors of omission in a row were excluded in the FFT (see Table 1). The data were not detrended in the SFAUS analysis, as the linear components of the RT variation over the run were of analytical interest.

In a separate test, the linear component in isolation was analysed by fitting regression lines to the RTs of each participant using a first order polynomial fit (linear). The slope of the regression line was then calculated.

Statistics: All dependent variables were calculated per participant and averaged per group for the Fixed and Random SARTs. The number of errors of commission and omission, mean RT and S.D. of RT were analysed in a Group (ADHD versus HFA versus Control) by Half (first half of trial versus second half) by SART (Fixed versus Random) three-way mixed factorial ANOVA design. The FFAUS was analysed in a Group by Half two-way mixed factorial ANOVA design for the Fixed and Random SARTs separately, to ensure the largest number of participants in each analysis. This was due to the exclusion criteria of the FFAUS (see above). The SFAUS and slope of regression line were analysed in a Group by SART two-way mixed factorial ANOVA. The alpha level was set at 0.05 and Bonferroni adjustments were used throughout the analysis.

## 3 Results

The ADHD group (mean 79, S.D. 6) scored significantly more highly on the Conners’ ADHD Index than the HFA (mean 66, S.D. 12) (*p* < 0.001) and control groups (mean 45, S.D. 5) (*p* < 0.001), [*F*(2,57) = 101.0, *p* < 0.001]. The HFA and control groups also differed significantly (*p* < 0.001). The HFA group (mean 5.0, S.D. 0.9) scored significantly more highly on the ASDI than the ADHD (mean 0.9, S.D. 1.0) (*p* < 0.001) and control groups (mean 0.1, S.D. 0.2) (*p* < 0.001), [*F*(2,58) = 197.0, *p* < 0.001]. The ADHD and control groups also differed significantly (*p* < 0.01). The higher than normal ratings of the ADHD and HFA groups on the ASDI and Conners’ ADHD Index, respectively, suggest that these groups may share some common symptoms. The significantly greater scores of the two groups on their respective symptom-rating scales nevertheless suggest that the two groups are distinct.

### 3.1 Commission errors

**Fig. 3:**
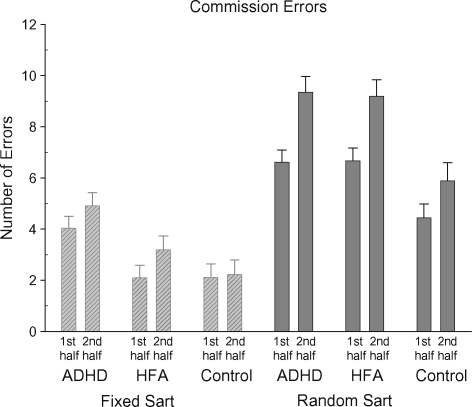
Mean commission errors (with standard errors) for the Fixed and Random SARTs for each participant group for the first and second halves of the task.

A significant Group and a significant SART version main effect were further explained by a significant Group by SART interaction, [*F*(2,59) = 5.39, *p* < 0.007] (see Fig. 3). On the Fixed SART, the ADHD group (mean 9.2, S.D. 5.6) made significantly more commission errors than the control (mean 4.4, S.D. 2.9) (*p* < 0.002) or HFA groups (mean 5.3, S.D. 3.4) (*p* < 0.011). There was no significant difference between the HFA and control groups. On the Random SART, the HFA group (mean 16.3, S.D. 5.2) made as many commission errors as the ADHD group (mean 16.8, S.D. 5.2). The control group (mean 10.5, S.D. 3.6) made significantly less errors than either the ADHD (*p* < 0.01) or HFA (*p* < 0.01) groups. The increase in commission errors in the Random SART by the HFA group was driving this interaction. All groups made significantly more errors on the Random SART compared with the Fixed SART. A significant Half main effect was further explained by a significant Half by SART interaction, [*F*(1,59) = 15.76, *p* < 0.001]. More commission errors were made in the second half of the Fixed (mean 3.5, S.D. 2.6) and the Random SARTs (mean 8.3, S.D. 3.3) compared with the first half of the Fixed (mean 2.8, S.D. 2.4) and Random SARTs (mean 6.0, S.D. 2.5). More commission errors were made in the Random SART compared with the Fixed SART during both the first and second halves of the task.

### 3.2 Omission errors

**Fig. 4:**
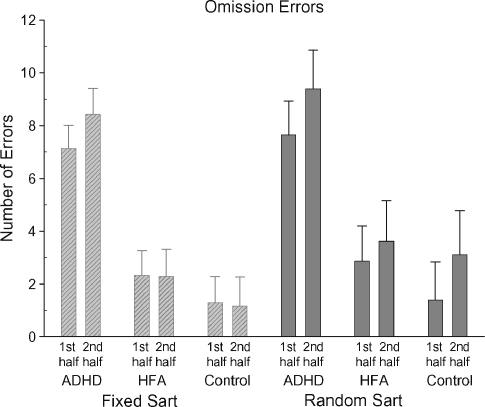
Mean omission errors (with standard errors) for the Fixed and Random SARTs for each participant group for the first and second halves of the task.

The ADHD group (mean 16.0, S.D. 11.0) (*p* < 0.0001) made significantly more omission errors than the HFA group (mean 4.8, S.D. 6.5) (*p* < 0.001) and the control group (mean 2.6, S.D. 2.4) across both the Fixed and Random SARTs [*F*(2,59) = 15.679, *p* < 0.001] (see Fig. 4). The HFA and control groups did not differ significantly. Across both SART versions, more omission errors were made in the second half of the task (mean 4.9, S.D. 6.7) compared with the first half (mean 4.0, S.D. 5.8), [*F*(1,59) = 4.56, *p* < 0.037]. The number of omission errors did not vary between the Fixed and Random SARTs.

### 3.3 Mean RT

**Fig. 5:**
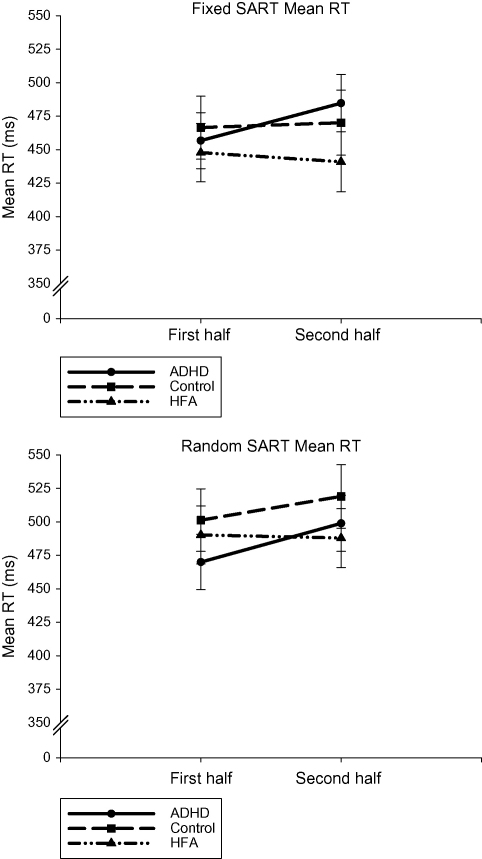
Mean response time (with standard errors) for the Fixed and Random SARTs for each participant group for the first and second halves of the task.

A significant Half main effect was further explained by a significant Half by Group interaction, [*F*(2,59) = 3.516, *p* < 0.036] (please see Fig. 5). The ADHD group (mean first half 463 ms, S.D. 93; mean second half 492, S.D. 93) significantly slowed in RT over the course of both the Fixed and Random SARTs. The control (mean first half 484 ms, S.D. 95; mean second half 495, S.D. 99) and the HFA groups (mean first half 469 ms, S.D. 111; mean second half 464, S.D. 115) maintained a consistent RT across both the SARTs. There was no difference in mean RT between the three groups during either the first or second halves. For all participants, the RT on the Random SART was slower (mean 493 ms, S.D. 99) compared with the Fixed SART (mean 461, S.D. 101), [*F*(1,59) = 8.352, *p* < 0.005].

### 3.4 Linear regression of RT

The linear regression of RT of the ADHD (mean 2.1, S.D. 7.1) was significantly greater than that of the HFA group (mean −1.0, S.D. 6.5), but did not vary significantly from the control group (mean −0.2, S.D. 4.2), [*F*(2,59) = 3.27, *p* < 0.045]. The HFA and control groups did not differ significantly. The positive slope of the regression indicated a slowing in RT over the course of the task for the ADHD group. There was no significant effect of the SART version.

### 3.5 Standard deviation of RT

A significant Half main effect was further explained by a significant Half by Group interaction, [*F*(2,59) = 5.25, *p* < 0.008]. The variability in RT of the ADHD group (mean S.D. first half 200, S.D. 74; mean S.D. second half 229, S.D. 74) significantly increased over the course of both the Fixed and Random SARTs. The control (mean S.D. first half 136 ms, S.D. 39; mean S.D. second half 149, S.D. 37) and the HFA groups (mean S.D. first half 154, S.D. 58; mean S.D. second half 156, S.D. 59) maintained a consistent S.D. of RT across both the Fixed and Random SARTs. The ADHD group was significantly more variable in RT than the control (*p* < 0.001) and HFA (*p* < 0.001) groups, for both the first and the second halves of the task. There was no significant difference in S.D. of RT between the control and HFA groups. The S.D. of RT did not vary between the Fixed and Random SARTs.

### 3.6 Slow-frequency area under the spectra

The average FFT spectrum for each group is shown in Fig. 2; the SFAUS is the area under the curve to the left of the dotted line. The SFAUS of the ADHD group (mean 831, S.D. 677) was significantly greater than that of the HFA (mean 425, S.D. 335) (*p* < 0.011) and control groups (mean 378, S.D. 229) (*p* < 0.007), [*F*(2,52) = 6.352, *p* < 0.003]. The HFA and control groups did not differ significantly. There was no significant effect of SART version on the SFAUS.

### 3.7 Fast-frequency area under the spectra—fixed SART

**Fig. 6:**
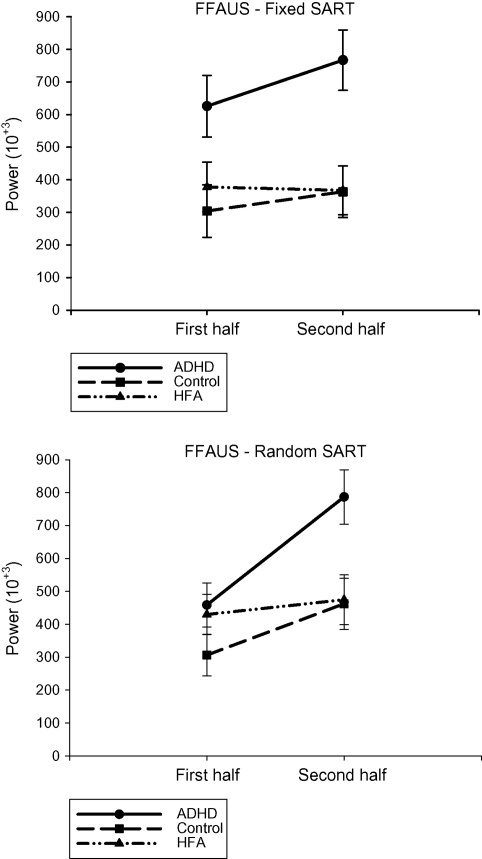
Fast-frequency Area Under Spectra scores (with standard errors) for each participant group for the first and second halves of the Fixed and Random SARTs.

The FFAUS is the area under the curve to the right of the dotted line in Fig. 2. The ADHD group (mean 696,265; S.D. 544,577) was significantly more variable in terms of moment-to-moment variability than the control group (mean 333,657; S.D. 167,110) (*p* < 0.004) and the HFA group (mean 372,557; S.D. 263,678) (*p* < 0.009), [*F*(2,48) = 6.753, *p* < 0.003] (see Fig. 6). The control and HFA groups did not differ significantly. There was no significant Half main effect or a significant interaction.

### 3.8 Fast-frequency area under the spectra—random SART

A significant Group and a Half main effect were further explained by a significant Group by Half interaction, [*F*(2,50) = 3.932, *p* < 0.026] (please see Fig. 6). In the first half of the Random SART, there was no difference between the ADHD (mean 458,610; S.D. 223,597), control (mean 306,117; S.D. 151,668) or HFA (mean 429,702; S.D. 367,510) groups. In the second half, the ADHD group (mean 768,721; S.D. 436,102) was significantly more variable in the fast frequency domain than either the control group (mean 462,119; S.D. 229,476) (*p* < 0.028) or the HFA group (mean 474,451; S.D. 305,024) (*p* < 0.034). The control and HFA groups did not differ significantly. Both the ADHD (*p* < 0.001) and control (*p* < 0.022) groups significantly increased the fast moment-to-moment variability in RT from the first to the second halves of the Random SART, whereas the HFA group did not change.

## 4 Discussion

The lack of consensus in the literature as to whether children with ADHD or autism have sustained attention and/or response inhibition deficits may reflect differences in task characteristics. By manipulating the predictability of stimulus presentations in the current study, dissociation in error and RT performance was noted between children with ADHD, HFA and controls. The children with ADHD demonstrated clear deficits in response inhibition and sustained attention, as measured by the number of commission and omission errors, the S.D. in RT and the fast, moment-to-moment variability in RT. In addition, they demonstrated waning performance over the course of the task, suggestive of deficits in arousal levels. The children with HFA, in contrast, performed normally on every measure of the SARTs except for the large number of commission errors made on the Random SART. This suggests that children with HFA have intact sustained attention but deficient response inhibition. Since sustained attention is known to be sub-served by fronto-parietal networks of the right-hemisphere (Coull et al., 1998, 1996; Pardo et al., 1991), as has been demonstrated specifically in imaging studies using the present SART paradigm (Fassbender et al., 2004; O’Connor et al., 2004), our behavioural data is suggestive of greater dysfunction within these circuits in ADHD than in autism.

The children with ADHD made a greater number of commission errors compared with the children with HFA and control children on the Fixed SART, suggesting sustained attention deficits. In contrast, the HFA group performed comparably with controls, possibly by making use of the externally cued, regular and predictable pattern of the Fixed SART. It is suggested that the children with HFA made special use of the regularly recurring sequence of digits leading up to the no-go “3”, in a systematic way, possibly by utilising compensatory cognitive mechanisms. Schmitz et al. (2006) recently reported that adults with autism demonstrated normal behavioural performance on executive function tasks, but significantly increased brain activation in the frontal, insular and parietal brain regions. The recruitment of additional areas of the brain to aid in task performance may be occurring in this group of children with HFA, possibly through the use of the external stimuli provided by the Fixed SART.

With the unpredictable stimulus presentation of the Random SART, the children with HFA made as many commission errors as the ADHD group. Indeed, the similarity in the number of commission errors made by the two groups was striking. The nature of response inhibition deficits in participants with HFA appears to be task-dependent. The children with HFA may be demonstrating a response inhibition deficit when external cues are unavailable for use, as the Random SART has a greater response inhibition component, when compared with the Fixed SART (Fassbender et al., 2004). Response inhibition deficits have previously been shown in children with HFA, particularly if the task tests prepotent inhibition, such as when an alternative response is needed to the primed response. Examples include the circle-drawing task (Geurts et al., 2004), the oculomotor anti-saccade task (Luna, Doll, Hegedus, Minshew, & Sweeney, 2007), the oculomotor delayed-response task (Minshew, Luna, & Sweeney, 1999) and the Go/No-Go task (Ozonoff & McEvoy, 1994). Children with HFA may not necessarily show deficits in a non-primed response inhibition task, such as the Stroop (Goldberg et al., 2005) and the visually guided saccade task (Minshew et al., 1999). Response inhibition deficits are suggestive of prefrontal and possibly parietal cortex dysfunction (Garavan, Ross, & Stein, 1999).

The sustained attention deficit of the children with ADHD was clearly shown by the high number of omission errors, made both during the Fixed and Random SARTs. In contrast, the children with HFA and the control children made a similar, lower number of omission errors. All children showed an increase in omission errors as the task progressed, suggesting that a progressive decline in performance is normal at this age.

The ADHD group showed a particular diminution in RT performance over the course of both the Fixed and Random SARTs, which was not shown by either the HFA or the control groups. The children with ADHD slowed in RT over the two halves of the tasks, as demonstrated by the mean RT and the linear regression analyses. The slow frequency variability in RT was significantly higher for the ADHD group. In comparison, the children with HFA and the control group maintained a steady performance across the tasks. The ADHD group may be affected by declining arousal. The SART runs for 5.5 min, which is a considerably shorter period compared with traditional vigilance tasks in which subjects must sustain attention for 15 (Teichner, 1974), 30 (Mackworth, 1968) or even 60 min periods (Paus et al., 1997). Nevertheless it is noteworthy that time-on-tasks effects are apparent even over this relatively short task duration. These results imply that arousal deficits may be one key driver of the ubiquitous findings of RT variability in the ADHD literature. This interpretation is consistent with the hypoarousal (Satterfield, Cantwell, & Satterfield, 1974) and the Cognitive Energetic models of ADHD (Sergeant, 2005). EEG, PET and rCBF studies have all provided evidence of cortical hypoarousal in ADHD (e.g. Lou, Henriksen, & Bruhn, 1984; Lou, Henriksen, Bruhn, Borner, & Neilsen, 1989), possibly due to dysfunctional sub-cortical areas. For instance, EEG recordings of adolescents with ADHD have shown increased theta activity and reduced beta activity, indicating a continuation of increasing slow wave activity in ADHD (Lazzaro et al., 1999). A number of recent genetic studies have shown associations between ADHD and allelic variation in genes controlling neurotransmitter systems that regulate arousal, such as noradrenaline (Madras, Miller, & Fischman, 2005), serotonin (Sheehan et al., 2005) and corticotrophin (Winsky-Sommerer, Boutrel, & de Lecea, 2005). Whether slow variability indexes arousal levels and is able to index genetic susceptibility in ADHD will be an important question for future research to address.

The ADHD group performed the Fixed and Random SARTs with greater fast-frequency (moment-to-moment) variability in RT and S.D. of RT than the children with HFA and the control children. The children with HFA and the control children performed the two SARTs with a similar amount of variability. Interestingly, the children with ADHD and control children showed greater fast-frequency variability in the second half of the Random SART compared with the first half, suggesting a time-on-task effect during this more difficult task. The HFA group, in both the Fixed and Random SARTs, appeared to have the capacity to maintain consistent fast-frequency variability in RT over the course of the task. Fast-frequency variation in RT likely reflects lapses in top-down attentional control that occur over relatively short time frames, up to the period of one SART cycle (13 s). There are suggestions that the areas of the fronto-parietal circuit are refreshed over time-spans of between 10 and 40 s (Parasuraman, Warm, & See, 1998; Pardo et al., 1991; Whitehead, 1991). The ADHD group's greater fast-frequency variability suggests fluctuating top-down attentional control, which in a harder task is also subject to time-on-task effects. Deficits in sustained attention in ADHD may reflect dysfunction of this executive control system (Silk et al., 2005; Sowell et al., 2003). The children with HFA did not demonstrate this greater fluctuation in fast-frequency variability, suggesting that they do not have deficits in top-down attentional control.

The ability to sustain attention to a routine task is an important aspect of executive control. There may be at least two different processes involved in the behavioural manifestation of deficient sustained attention: a gradual deterioration in attention to a task and a fast phasic variation in top-down attentional control. An incremental worsening in attention to a task, as reflected by slow-frequency variability in RT (SFAUS), may be linked to a deficit in brain arousal levels, related to the functioning of sub-cortical structures (locus coeruleus, pulvinar, the basal forebrain, thalamus, brain stem reticular formation), the anterior cingulate and neurotransmitter dysfunction (noradrenergic, cholinergic and serotonergic) (Biederman & Spencer, 1999; Moruzzi & Magoun, 1949; Paus, 2001; Paus et al., 1997). These systems might be more dysfunctional in ADHD than in HFA, especially if the task is endogenously taxing. Top-down attentional control may also wax and wane throughout the course of a task in a phasic fashion, affecting the ability to maintain concentration on a task. Sustained attention is thought to reflect the activity of the right-lateralised fronto-parietal attentional networks (Manly et al., 2003), which may be affected in ADHD to a far greater degree than in HFA. There may be a multisecond oscillatory cycle of sustained attention that is linked to physiological processes such as basal ganglia neuronal activity and cerebral hemodynamic response (Castellanos et al., 2005). Future research will need to determine the neural substrates of these two distinct processes and their functioning in ADHD and HFA and control children. In addition, it would be interesting to investigate how these two proposed processes vary in the sub-groups of ADHD, to enable a greater understanding of the heterogeneity of this disorder.

The clear distinction in performance between the children with ADHD and HFA on the Fixed and Random SARTs suggests that sustained attention may not be a deficit shared by the two disorders. Response inhibition may be a shared feature of the two disorders, especially in tasks when external cues that provide some structure are unavailable for children with HFA. These clear findings highlight the potential usefulness of CPT-like tasks in the assessment and conceptualisation of children with psychiatric disorders. The response inhibition, sustained attention and arousal deficits of children with ADHD should be addressed when designing cognitive behavioural therapies. The usefulness of external cues in providing a structure for cognition in children with HFA has been highlighted in this comparison of performance on the Fixed and Random SARTs.

This study is limited by the retrospective use of the CPRS-R:S and the ASDI for a sub-group of the children and this must be taken into account when drawing conclusions from the data. It is important to note that 12 (57%) children with HFA scored at least 65 on the Conners’ ADHD Index. Whilst the HFA and ADHD groups differed significantly in their mean ADHD Index scores, some of the children with HFA displayed clinically-relevant ADHD-like behaviours, highlighting the potential comorbidity of these two disorders and the heterogeneity of the HFA group.

In summary, four key findings resulted from the present study. First, the ADHD group showed pervasive deficits in sustained attention in both the Fixed and Random SARTs, particularly when the requirements for endogenous control of attention were high. The clear dissociation in performance of the ADHD group, compared with the HFA and control groups, highlights the potential utility of the SART measures as specific endophenotypes for ADHD. Second, the ADHD group demonstrated a time-on-task effect over the course of the Fixed and Random SARTs, as reflected in the increased slow-frequency variability, mean RT and linear regression of RT, possibly reflecting a decrease in arousal levels. That we found deficits for slow-frequency variability in the ADHD group but not in the HFA group also suggests that variability is not a simple consequence of cerebral disruption (see also MacDonald et al., 2006; Stuss et al., 2003). Slow-frequency variability in ADHD, rather, may be a consequence of specific disruption within the arousal system. This hypothesis awaits confirmation with neuroimaging. Third, the ADHD and HFA groups both showed heightened levels of commission errors on the Random SART, compared with the control group, suggesting deficits in response inhibition. Interestingly, the finding of a response inhibition deficit in the HFA group that was of comparable effect size to the ADHD group, adds to a burgeoning literature suggesting inhibitory deficits in HFA. Fourth, the HFA group made a normal number of commission errors on the Fixed SART, possibly by utilising the predictable sequence of digits preceding the NoGo “3” stimulus as cues, which is suggestive of a systematizing aptitude. This study provides detailed behavioural evidence of significantly greater sustained attention dysfunction in ADHD than in HFA. This behavioural deficit in ADHD may be underpinned by greater dysfunction within fronto-parietal areas and the subcortical arousal system.

## Figures and Tables

**Table 1:** Information on the ADHD, HFA and Control children

Group	ADHD	HFA	Control
Number	23	21	18
Age (mean, S.D.)	10.5 (2.4)	12.2 (2.4)	11.1 (1.9)
IQ (mean, S.D.)	98.7 (14.6)	97.3 (12.3)	107.7 (11.6)
Left-handers	4	2	0
Number of Conners’ Parental responses	23	21	18
Conners’ ADHD Index (mean, S.D.)	78.8 (6.2)^*^^	65.5 (11.6)^*#^	45.0 (4.8)^^#^
Conners’ Hyperactive Subscale (mean, S.D.)	84.5 (5.8)^*^^	67.0 (13.6)^*#^	47.1 (7.7)^^#^
Conners’ Restless/Impulsive Subscale (mean, S.D.)	76.9 (8.2)^*^^	64.0 (11.4)^*#^	44.6 (4.8)^^#^
Number of ASDI Parental responses	23	21	17
ASDI Total score (mean, S.D.)	0.87 (1.0)^*^^	5.0 (0.9)^*#^	0.06 (0.2)^^*^
No. included in Fixed SART FFAUS analysis	13	20	18
No. included in Random SART FFAUS analysis	16	19	18
No. included in Fixed & Random SART SFAUS analysis	17	20	18

ADHD: Attention deficit hyperactivity disorder; HFA: high-functioning autism; IQ: intelligence quotient; ASDI: Asperger Syndrome Diagnostic Interview; SART: Sustained Attention to Response Task; FFAUS: fast-frequency area under the spectra; SFAUS: slow-frequency area under the spectra; ^*^significant difference between ADHD and HFA; ^∧^significant difference between ADHD and controls; ^#^significant difference between HFA and controls; alpha level set at 0.05.

## Footnotes

1 One child was taking 0.5 ml Risperidol per day. One child was taking 30 mg Cipramil per day.

2 Hamming-windowing and zero-padding are standard preliminary steps in calculating FFTs and are explained in text books on time-series analyses, e.g. (Oppenheim, Schafer, & Buck, 1999).
